# Interpretable EOG-based gaze classification using tree-based models: A SHAP and LIME analysis

**DOI:** 10.1371/journal.pone.0361971

**Published:** 2026-10-29

**Authors:** Ayse Nur Ay Gul

**Affiliations:** 1 Department of Mechatronics Engineering, Sakarya University of Applied Sciences, Sakarya, Turkey; 2 Biomedical Technologies Application and Research Center (BIYOTAM), Sakarya University of Applied Sciences, Sakarya, Turkey; Daffodil International University, BANGLADESH

## Abstract

Electrooculography (EOG) is a low-cost biosignal modality for gaze-based human–computer interaction, but reliable validation and model interpretability remain important challenges for machine learning-based gaze classification. This study proposes an explainable EOG gaze classification framework using UNIOVI-EOG dataset. Horizontal and vertical EOG recordings from nine participants were converted into 100 ms non-overlapping windows, and only stable windows with at least 90% label purity were retained. Time-domain window features were extracted and evaluated using Decision Tree, Random Forest, XGBoost, and CatBoost classifiers under subject-wise five-fold cross-validation, ensuring that participants in the validation folds were not included in training. The final dataset contained 81,481 accepted windows across nine gaze classes. All models achieved very high subject-wise performance, with Random Forest providing the strongest overall results. The high performance was consistent with the controlled fixation protocol and the strong separability of horizontal and vertical EOG amplitude patterns, as supported by simple mean-amplitude baseline and leakage diagnostic analyses. Model interpretability was assessed using SHapley Additive exPlanations (SHAP) and Local Interpretable Model-Agnostic Explanations (LIME). The explanations showed that amplitude-related features, especially channel means, medians, quantiles, and absolute mean values, were the main contributors to classification decisions. SHAP–LIME rank correlation and permutation-based faithfulness analysis provided additional quantitative support for the explanations. These findings suggest that leakage-controlled, window-level, explainable machine learning can provide accurate and physiologically interpretable EOG gaze classification in controlled experimental settings.

## 1. Introduction

Electrooculography (EOG) is a non-invasive method used to record eye movements by measuring the potential difference between the cornea and retina. Due to its simplicity, low cost, and robustness in various environments, EOG is widely used in biomedical and assistive technologies [1,2]. Common applications include human–computer interface (HCI) systems, robotic arm control, and eye-based typing, where user intent is captured through gaze direction or blink detection [3–5]. Because EOG signals are typically low-dimensional and structured, they are well-suited for machine learning (ML)-based classification.

EOG-based systems have been applied in a wide range of assistive and human–computer interaction applications, including gaze estimation, communication interfaces, and real-time control systems [6,7]. As the use of EOG signals in practical applications continues to increase, not only classification performance but also the reliability and transparency of machine learning models have become important considerations. In biomedical and assistive technologies, understanding how a model reaches a particular decision is often as important as the prediction itself, particularly when these decisions may affect user interaction or system behavior [8–11].

To improve the accuracy and reliability of EOG classification, various ML models have been employed [12]. Tree-based algorithms such as decision tree (DT), random forest (RF), eXtreme gradient boosting (XGBoost) and categorical boosting (CatBoost), are especially popular due to their efficiency, strong performance, and ability to handle non-linear data patterns [13–16]. RF uses ensemble learning to reduce overfitting, while boosting models such as XGBoost, and CatBoost iteratively refine predictions to improve accuracy [16–18].These models also offer built-in feature importance measures, making them useful in physiological signal classification where some features may be noisy or redundant.

However, despite their predictive power, these models are often seen as “black boxes” due to the complexity of their internal structures. This lack of interpretability limits their adoption in sensitive domains such as healthcare and assistive systems, where model decisions may directly impact safety and user experience. To address this, the field of explainable artificial intelligence (XAI) has gained attention. XAI techniques aim to provide meaningful insights into how a model arrives at its predictions, supporting transparency, trust, and system validation [19,20]. In biosignal research, XAI has been used to interpret ML models trained on electroencephalography (EEG), electrocardiography (ECG), and electromyography (EMG) data, helping to validate model logic and discover important signal features [9–11].

Two widely used model-agnostic XAI methods are SHapley Additive exPlanations (SHAP) and Local Interpretable Model-Agnostic Explanations (LIME). SHAP is based on game theory and provides consistent and theoretically grounded explanations by estimating each feature’s contribution to a model’s output [21,22]. LIME, in contrast, builds local surrogate models to explain individual predictions by perturbing input features [23]. Both methods have been applied to biomedical signal classification, offering insights into model behavior and improving interpretability [24,25]. However, despite the structured nature of EOG signals and their increasing use in assistive control systems, SHAP and LIME remain relatively underexplored in EOG-based classification.

Recent studies in biosignal analysis have shown that evaluation protocols can substantially influence reported classification performance [26]. In particular, subject overlap between training and testing data or the use of temporally adjacent samples may lead to overly optimistic results. Although high classification accuracies have been reported in many EOG studies, fewer studies have investigated model interpretability together with validation strategies designed to reduce the risk of data leakage and improve generalizability [27–29].

Most existing studies on EOG classification focus on achieving high accuracy but provide little to no insight into how models make decisions [4,5,15]. As EOG is increasingly used in real-world interfaces, there is a growing need for interpretable models that can guide system development, detect model errors, and build user trust. The lack of explainability research in this area represents a clear gap in the current literature.

This study addresses this gap by applying several tree-based classifiers DT, RF, XGBoost and CatBoost to EOG signal classification. SHAP and LIME are used to explain model behavior and identify the most influential features. The results are analyzed to compare the usefulness of these two explanation methods, and to explore which eye positions are easiest or hardest to classify. This work aims to support the development of interpretable and reliable EOG-based biomedical systems.

## 2. Materials and Methods

The overall pipeline proposed in this work is presented in Fig 1.

**Fig 1:**
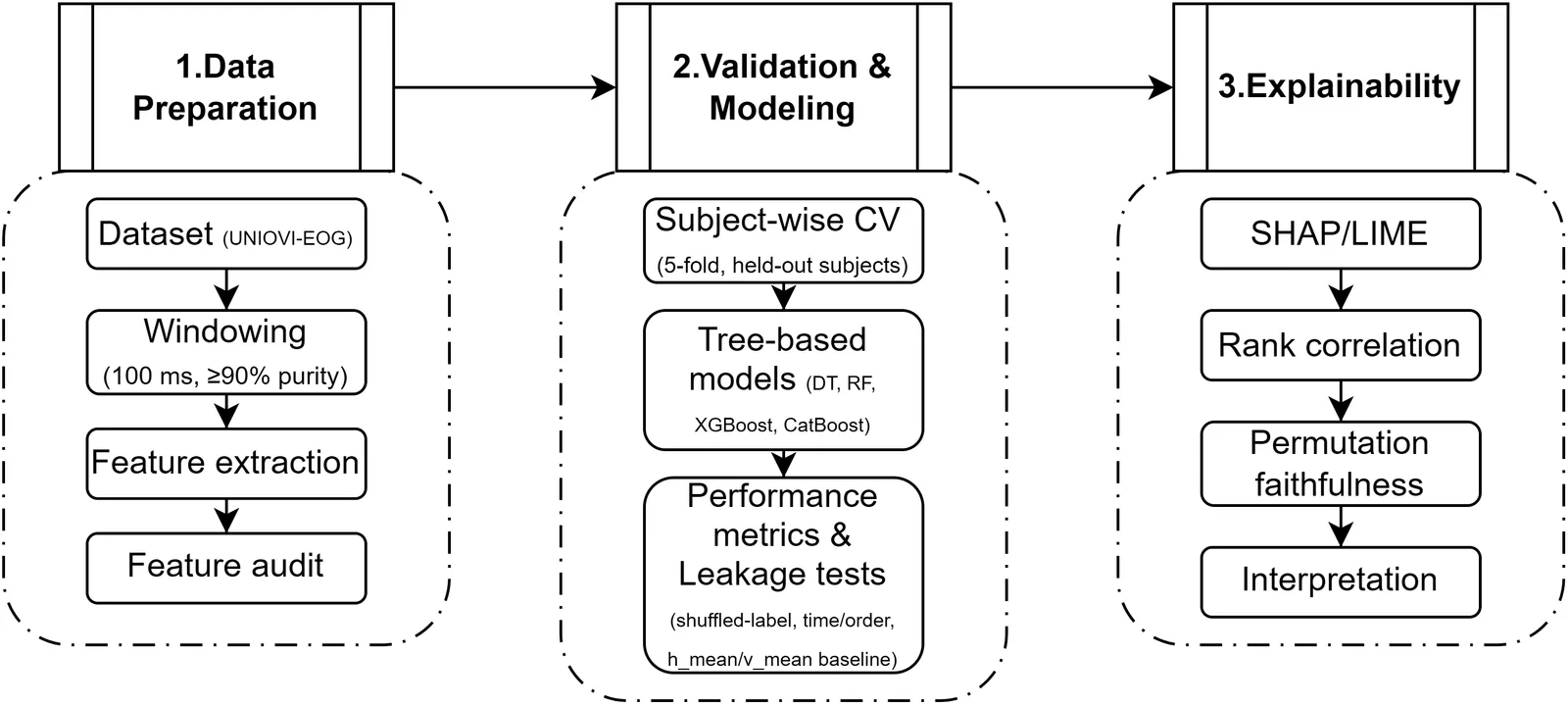
Proposed AI/XAI workflow diagram.

The pipeline includes dataset loading, preprocessing and preparation, model training, performance evaluation, validation of the results, and assessment of the extracted features using explainable artificial intelligence (XAI) algorithms. The figure also illustrates how the contribution of each feature was analyzed in relation to the final model decisions. All steps were performed within a structured evaluation framework to maintain the consistency, transparency, and reliability of the results.

### 2.1. Dataset

This study used the publicly available UNIOVI-EOG dataset, which contains electrooculography (EOG) recordings acquired during controlled gaze-direction tasks. The dataset includes recordings from nine healthy adult participants who performed predefined visual fixation tasks toward nine screen positions. Horizontal and vertical bipolar EOG channels were recorded at a sampling frequency of 1 kHz. Each raw data file contained four main columns: the horizontal EOG signal (h_signal), the vertical EOG signal (v_signal), the horizontal gaze label (h_label), and the vertical gaze label (v_label) [27].

The gaze labels were defined using two directional components. The horizontal component represented left, center, and right gaze positions, while the vertical component represented downward, center, and upward gaze positions. Each component was encoded using one of three values: −1, 0, or 1. The horizontal and vertical labels were combined into a single multiclass target label in the form h_label_v_label. This resulted in nine gaze classes corresponding to the possible combinations of horizontal and vertical gaze directions: −1_-1, −1_0, −1_1, 0_-1, 0_0, 0_1, 1_-1, 1_0, and 1_1.

During data loading, participant and session identifiers were preserved for validation and auditing purposes. However, these metadata were not used as model input features. They were retained only to ensure subject-wise data splitting and to verify that no participant appeared simultaneously in the training and validation subsets.

### 2.2. Window-level sample construction

To avoid treating adjacent time points as independent observations, the raw EOG recordings were transformed into window-level samples [6,30]. For each participant and session, consecutive EOG samples were grouped into fixed-length windows. A window size of 100 samples was used, corresponding to 100 ms of signal at the 1 kHz sampling rate. Non-overlapping windows were generated using a step size of 100 samples.

Each window was assigned a gaze label according to the majority class within that window. To reduce the influence of transition periods between gaze targets, a label-purity criterion was applied. A window was accepted only when at least 90% of its samples belonged to the same majority class. Windows that did not meet this threshold were excluded from further analysis. This procedure ensured that each retained window represented a stable gaze state rather than a transition between gaze directions [31].

The use of window-level samples provides a more realistic representation of EOG-based gaze classification than single time-point classification, because gaze direction is inferred from sustained signal patterns rather than isolated instantaneous amplitude values [28].

### 2.3. Feature extraction

For each accepted window, descriptive time-domain features were extracted separately from the horizontal and vertical EOG channels. These features were selected to summarize the amplitude level, dispersion, temporal trend, and energy of the EOG signal within each window [32].

For both the horizontal and vertical channels, the following features were calculated: mean, standard deviation, minimum, maximum, median, 25th percentile, 75th percentile, interquartile range, range, mean absolute amplitude, energy, linear slope, first value, last value, and signal change between the first and last samples. In addition, cross-channel features were computed to describe the relationship between the horizontal and vertical EOG components. These included the correlation between the two channels, the mean horizontal–vertical difference, the mean absolute horizontal–vertical difference, and the horizontal-to-vertical energy ratio.

Only signal-derived features were included in the model input matrix. Metadata such as participant identifier, session identifier, sample index, window start and end positions, number of samples per window, label purity, and class labels were excluded from the feature matrix. This strict feature selection was applied to prevent any direct or indirect leakage of participant, time-order, or label-related information into the models.

### 2.4. Subject-wise validation strategy

Model performance was evaluated using subject-wise cross-validation. In this validation design, all samples from a given participant were assigned exclusively to either the training subset or the validation subset within each fold. Therefore, no participant appeared in both training and validation data during the same fold. This strategy was used to estimate model generalization to unseen participants more conservatively and to reduce the risk of inflated performance caused by subject-specific information being shared across training and validation sets [26].

A five-fold grouped cross-validation procedure was applied. In each fold, the model was trained using data from the training participants and evaluated on held-out participants that were not seen during training. Fold assignments were generated using participant identifiers as grouping variables. The absence of subject overlap between the training and validation subsets was verified for every fold.

This subject-wise validation protocol was used as the primary evaluation strategy throughout the study. Results obtained from the final model trained on the complete dataset were not used for validation reporting.

### 2.5. Machine learning models

Four tree-based machine learning classifiers were evaluated for multiclass EOG gaze classification: Decision Tree, Random Forest, eXtreme Gradient Boosting (XGBoost), and Categorical Boosting (CatBoost). These models were selected because tree-based classifiers are well suited for structured tabular data and can model nonlinear relationships between EOG-derived features and gaze classes [33].

Fixed hyperparameter settings were used for all models to ensure a consistent and reproducible comparison across the same subject-wise validation folds. The Decision Tree classifier was trained with balanced class weighting, no maximum depth restriction, and a minimum leaf size of one. The Random Forest model used 300 trees, balanced subsampling, and square-root feature selection at each split. XGBoost was trained using 500 estimators, a maximum tree depth of 4, a learning rate of 0.05, row subsampling of 0.80, column subsampling of 0.80, a multiclass soft-probability objective, and multiclass log-loss evaluation. CatBoost was trained using 500 iterations, a depth of 6, a learning rate of 0.05, and a multiclass loss function. Class balancing was applied during model training to reduce the possible influence of class imbalance. No nested hyperparameter optimization was performed; therefore, the model comparison should be interpreted as a comparison under fixed, predefined settings rather than as a fully optimized benchmark.

The same validation folds were used for all models to ensure a fair comparison. No participant-level metadata or time-order variables were provided to the classifiers.

### 2.6. Performance evaluation

Model performance was assessed on the held-out subjects in each cross-validation fold. The primary evaluation metrics included accuracy, balanced accuracy, macro-averaged F1 score, weighted F1 score, macro-averaged precision, macro-averaged recall, and Matthews correlation coefficient. Macro-averaged metrics were included because the accepted window-level dataset showed moderate class imbalance across the nine gaze classes. Balanced accuracy and Matthews correlation coefficient was also reported to provide additional robustness beyond overall accuracy.

For each model, performance metrics were calculated separately for each fold and then summarized as mean and standard deviation across folds. Confusion matrices and class-wise classification reports were also generated to examine the distribution of errors across gaze classes.

### 2.7. Leakage and sanity-check analyses

Additional diagnostic analyses were performed to evaluate whether the observed classification performance could be explained by unintended leakage or protocol-related artifacts. First, a shuffled-label sanity check was conducted by randomly permuting the class labels and repeating model evaluation. Under this condition, model performance was expected to decrease toward chance level for the nine-class classification task. High performance after label shuffling would indicate possible leakage; therefore, this test was used as an internal control.

Second, a time/order-only baseline was evaluated using only time-related metadata, such as window start and end positions, where available. These variables were not used in the final classification models. This diagnostic test was used to determine whether the experimental order alone could predict the gaze classes. The results of this baseline were compared with the performance of the signal-derived feature models.

Third, a simple physiological baseline using only the mean horizontal and vertical EOG amplitudes was evaluated. This baseline was included to determine whether the gaze classes were separable primarily from the average amplitude levels of the two EOG channels. This analysis helped contextualize the performance of the more complex tree-based models.

### 2.8. Explainable artificial intelligence analysis

Explainable artificial intelligence analysis was performed to interpret the decision behavior of the trained tree-based models. Two complementary explanation methods were used: SHapley Additive exPlanations (SHAP) and Local Interpretable Model-Agnostic Explanations (LIME).

SHAP analysis was used to estimate the global and class-specific contribution of each window-level EOG feature to model predictions. For tree-based models, SHAP values were computed on held-out validation samples from the subject-wise cross-validation procedure. Mean absolute SHAP values were used to quantify overall feature importance, while signed SHAP values were used to examine the direction of feature influence for specific gaze classes.

LIME analysis was used to provide local explanations for individual held-out validation instances. For selected correctly classified samples, LIME generated local feature weights by approximating the model behavior around each instance using an interpretable surrogate model. These explanations were used to examine whether local decision patterns were consistent with the global SHAP-based feature attributions.

To provide a quantitative comparison between SHAP and LIME, feature-importance rankings obtained from both methods were compared using Spearman rank correlation. This analysis assessed whether the two explanation methods identified similar influential features. In addition, permutation-based feature faithfulness analysis was performed by perturbing selected important features and measuring the resulting decrease in macro-F1 score. This analysis was used to evaluate whether features identified as important by the explanation methods had a measurable effect on predictive performance.

## 3. Results

### 3.1. Window-level dataset and class distribution

After applying the window-level sample construction procedure, a total of 81,481 accepted EOG windows were retained for analysis. These windows represented stable gaze states that satisfied the predefined label-purity threshold. The accepted windows were distributed across the nine gaze classes, as shown in Table 1.

**Table 1:** Window-level class distribution after label-purity filtering.

Class label	Number of windows	Percentage (%)
**0_0**	16805	20.62
**1_-1**	10938	13.43
**−1_-1**	9302	11.41
**−1_1**	9085	11.15
**−1_0**	8232	10.10
**1_0**	7737	9.50
**0_-1**	7387	9.10
**1_1**	6771	8.31
**0_1**	5224	6.41

The class distribution showed moderate imbalance. The center gaze class (0_0) was the most frequent class, with 16,805 windows, corresponding to 20.62% of the dataset. The least frequent class was 0_1, with 5,224 windows, corresponding to 6.41% of the dataset. The remaining classes ranged from 6,771–10,938 windows. Because of this imbalance, model evaluation was not based on accuracy alone; balanced accuracy, macro-F1 score, macro-precision, macro-recall, weighted-F1 score, and Matthews correlation coefficient was also reported.

### 3.2. Subject-wise validation and leakage diagnostics

The evaluation protocol used subject-wise cross-validation to ensure that samples from the same participant did not appear simultaneously in training and validation subsets. Across the five folds, the validation set contained either one or two held-out participants, while the remaining participants were used for training. No subject overlap was observed between the training and validation subsets in any fold.

To further examine whether the classification results could be explained by unintended leakage or protocol-related artifacts, two diagnostic analyses were performed. In the shuffled-label sanity check, the class labels were randomly permuted before model training. Under this condition, the Decision Tree model achieved a mean accuracy of 0.123 and a mean macro-F1 score of 0.110, which is close to chance-level performance for a nine-class classification task. This result indicates that the classification pipeline did not maintain high performance when the relationship between EOG features and gaze labels was disrupted.

The time/order-only baseline produced a mean accuracy of 0.243 and a mean macro-F1 score of 0.199. Although this result was above chance level, it remained substantially lower than the performance of the EOG-feature-based models. This suggests that time/order information alone was not sufficient to explain the high classification performance. In contrast, a simple physiological baseline using only the mean horizontal and vertical EOG amplitudes achieved perfect classification performance across all folds. This finding indicates that, in this controlled dataset, gaze classes were highly separable using the average amplitude levels of the two EOG channels.

In Table 2. Shuffled-label testing, time/order-only classification, and a simple mean-amplitude baseline were used to evaluate whether the observed performance could be explained by label leakage, temporal order, or strong physiological separability of the EOG channels.

**Table 2:** Diagnostic analyses for leakage and protocol-related effects.

Diagnostic analysis	Accuracy, mean ± SD	Balanced accuracy, mean ± SD	Macro-F1, mean ± SD
Shuffled-label sanity check	0.123 ± 0.005	0.112 ± 0.004	0.110 ± 0.005
Time/order-only baseline	0.243 ± 0.049	0.209 ± 0.032	0.199 ± 0.035
Simple h_mean/v_mean baseline	1.000 ± 0.000	1.000 ± 0.000	1.000 ± 0.000

The shuffled-label sanity check produced near-chance performance, indicating that the model did not maintain high accuracy when the relationship between EOG features and gaze labels was removed. The time/order-only baseline also produced much lower performance than the EOG-feature-based models, suggesting that temporal order or window position alone did not explain the classification results. In contrast, the simple h_mean/v_mean baseline achieved perfect performance, showing that the gaze classes in this controlled dataset were highly separable using basic horizontal and vertical EOG amplitude information.

### 3.3. Subject-wise classification performance

The performance of the four tree-based classifiers under subject-wise cross-validation is summarized in Table 3. All models achieved high classification performance on held-out subjects. Random Forest achieved the highest performance, with mean values of 1.000 for accuracy, balanced accuracy, macro-F1 score, weighted-F1 score, macro-precision, macro-recall, and Matthews correlation coefficient across the five folds.

**Table 3:** Subject-wise classification performance of the evaluated tree-based models.

Model	Accuracy	Balanced accuracy	Macro-F1	Weighted-F1	MCC
Random Forest	1.000 ± 0.000	1.000 ± 0.000	1.000 ± 0.000	1.000 ± 0.000	1.000 ± 0.000
Decision Tree	0.999 ± 0.000	0.999 ± 0.000	0.999 ± 0.000	0.999 ± 0.000	0.999 ± 0.000
CatBoost	0.999 ± 0.000	0.999 ± 0.000	0.999 ± 0.000	0.999 ± 0.000	0.999 ± 0.000
XGBoost	0.999 ± 0.000	0.999 ± 0.000	0.999 ± 0.000	0.999 ± 0.000	0.999 ± 0.000

Decision Tree also achieved near-perfect performance, with a mean accuracy of 0.99999 and a mean macro-F1 score of 0.99999. CatBoost achieved a mean accuracy of 0.99994 and a mean macro-F1 score of 0.99993, while XGBoost achieved a mean accuracy of 0.99985 and a mean macro-F1 score of 0.99982. The small standard deviations across folds indicate stable classification performance across held-out participants.

Although all models performed strongly, the results should be interpreted in the context of the controlled experimental protocol and the strong separability of the window-level EOG amplitude features. The diagnostic baseline using only h_mean and v_mean suggests that the gaze classes in this dataset can be largely distinguished from the average horizontal and vertical signal amplitudes.

In Table 3. values are reported as mean ± standard deviation across five subject-wise validation folds. Accuracy, balanced accuracy, macro-F1, weighted-F1, and Matthews correlation coefficient was used to evaluate performance under the held-out participant validation protocol.

All evaluated tree-based models achieved near-perfect performance under the subject-wise validation protocol. Random Forest achieved the highest overall performance, with perfect mean scores across all reported metrics. Decision Tree, CatBoost, and XGBoost also produced extremely high and stable results, with very small standard deviations across folds. These findings indicate that the selected EOG features provided highly discriminative information for gaze-class classification in the controlled experimental setting.

### 3.4. Global SHAP feature importance

SHAP analysis was performed to identify the most influential window-level EOG features contributing to model predictions. For the Random Forest model, the highest global SHAP contributions were mainly associated with central tendency and amplitude-distribution features from the horizontal and vertical EOG channels. The most important features included h_q75, v_mean, v_q75, h_mean, h_median, v_q25, v_median, v_abs_mean, h_q25, and h_abs_mean.

These findings indicate that gaze classification was primarily driven by stable amplitude-level features rather than high-variability or cross-channel correlation features. Features such as h_std, v_std, h_range, v_range, h_iqr, v_iqr, and hv_corr showed comparatively low SHAP importance. This pattern is consistent with the controlled fixation nature of the dataset, where gaze direction is expected to be reflected mainly in sustained horizontal and vertical EOG amplitude shifts.

In Fig 2, mean absolute SHAP values show the overall contribution of each window-level EOG feature to model predictions on held-out validation samples. Higher values indicate stronger average influence on classification decisions.

**Fig 2:**
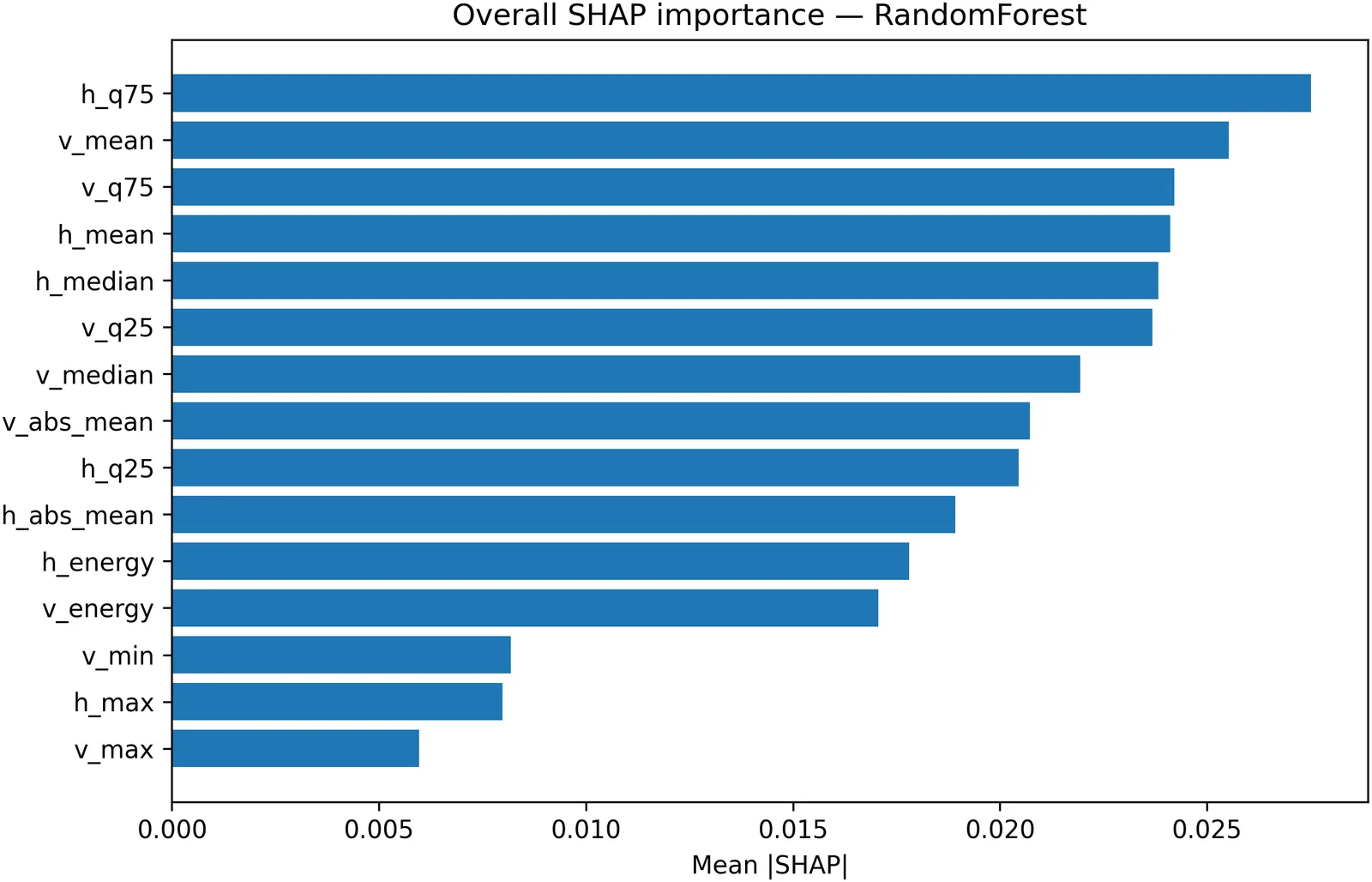
Overall SHAP feature importance for Random Forest.

Fig 3 shows that each point represents a held-out validation sample, with the horizontal position indicating the SHAP value and the color indicating the corresponding feature value. The plot illustrates how high and low feature values contributed to model predictions.

**Fig 3:**
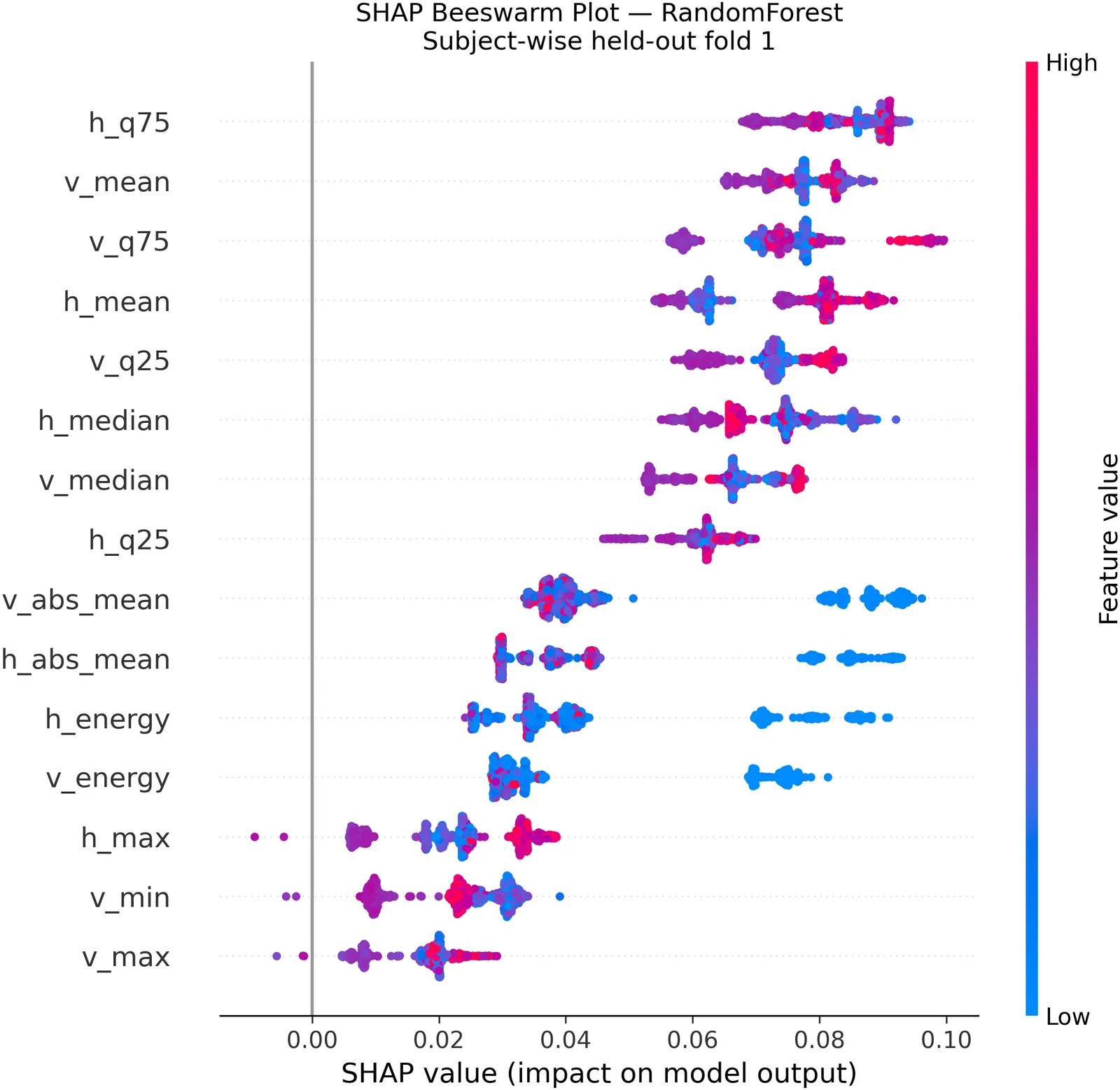
SHAP beeswarm plot for the Random Forest model.

### 3.5. Class-wise SHAP interpretation

Class-wise SHAP analysis was used to examine how feature contributions varied across the nine gaze directions. The class-level SHAP patterns showed that gaze classification was mainly governed by features derived from the horizontal and vertical EOG channels. Horizontal gaze classes were primarily associated with horizontal-channel amplitude features, while vertical and diagonal classes showed combined contributions from both horizontal and vertical features.

The class-wise feature-importance heatmap provides a compact overview of these relationships. Classes involving lateral gaze components showed stronger dependence on horizontal-channel features, whereas classes with vertical components showed increased contribution from vertical-channel features. Diagonal gaze classes were characterized by combined horizontal and vertical feature contributions, reflecting the two-dimensional nature of the gaze labels.

The center gaze class (0_0) was also separable from the directional gaze classes, mainly through differences in the amplitude-level features of both EOG channels. This is consistent with the expected physiological pattern that central fixation produces a different horizontal–vertical amplitude profile compared with directional gaze positions.

In Fig 4, the heatmap summarizes mean absolute SHAP values across gaze classes and features, showing how horizontal, vertical, and combined EOG features contributed differently to the nine gaze classes.

**Fig 4:**
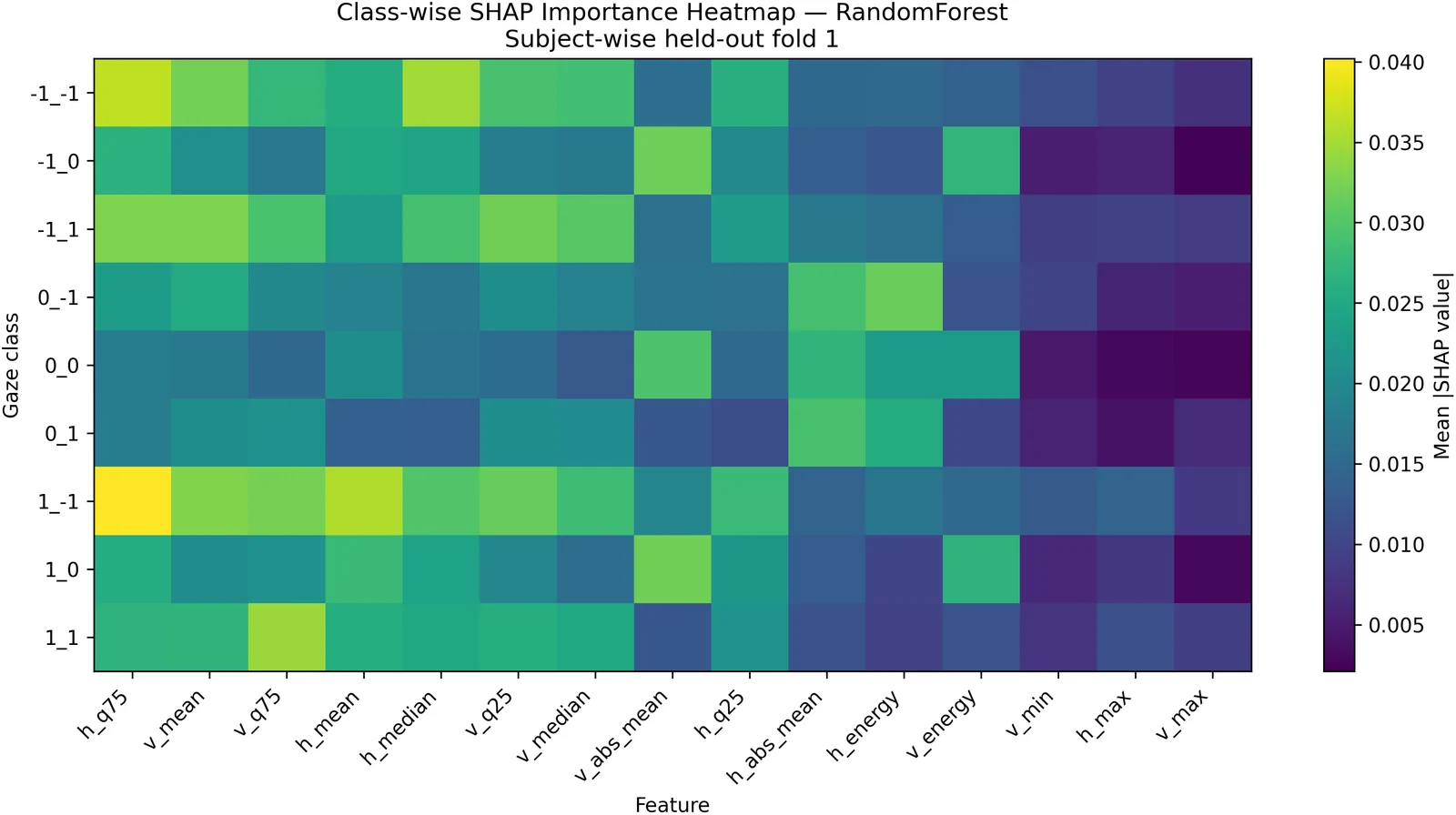
Class-wise SHAP importance heatmap for the Random Forest model.

### 3.6. LIME explanations and SHAP–LIME agreement

LIME was used to generate local explanations for selected correctly classified held-out validation samples. These local explanations were compared with SHAP-based feature rankings using Spearman rank correlation to assess the agreement between local and global explanation patterns. The SHAP–LIME rank-correlation results are summarized in Table 4.

**Table 4:** Agreement between SHAP and LIME feature-importance rankings.

Model	Number of common features	Spearman ρ	p-value
CatBoost	31	0.808	3.98 × 10^−8^
Decision Tree	31	0.242	0.190
Random Forest	31	0.827	1.01 × 10^−8^
XGBoost	31	0.721	4.76 × 10^−6^

Spearman rank correlation was calculated between SHAP-based and LIME-based feature rankings for each model using the common feature set. Higher positive values indicate stronger agreement between global and local explanation patterns.

The SHAP–LIME rank-correlation results showed strong and statistically significant agreement for Random Forest, CatBoost, and XGBoost, indicating that the local LIME explanations were generally consistent with the global SHAP feature-importance patterns. Random Forest showed the highest agreement, followed by CatBoost and XGBoost. In contrast, Decision Tree showed weak and non-significant agreement, suggesting lower explanation stability for the single-tree model.

### 3.7. Permutation-based feature faithfulness analysis

Permutation-based feature faithfulness analysis was performed to examine whether features identified as important had a measurable effect on classification performance. The effect of feature perturbation was quantified as the decrease in macro-F1 score after permuting selected features.

For the Decision Tree model, permutation of several important features resulted in substantial reductions in macro-F1 score. The largest decrease was observed after permuting v_energy, which reduced macro-F1 by 0.487. Other influential features included h_abs_mean, h_q75, v_min, and h_mean, which produced macro-F1 decreases of 0.340, 0.282, 0.227, and 0.149, respectively. These results indicate that the Decision Tree model relied strongly on a limited set of amplitude- and energy-related features.

For the ensemble models, the macro-F1 decrease after single-feature permutation was smaller. CatBoost showed a maximum macro-F1 decrease of 0.0008, while XGBoost showed a maximum decrease of 0.0015. For Random Forest, the tested single-feature perturbations did not produce a measurable decrease in macro-F1. This does not necessarily indicate that the features were unimportant; rather, it suggests that the ensemble models were robust to single-feature perturbation, likely because multiple correlated window-level amplitude features carried redundant class-discriminative information.

Overall, the faithfulness analysis supports the importance of amplitude- and energy-related EOG features, while also showing that feature redundancy should be considered when interpreting permutation-based importance in highly separable window-level datasets.

In Table 5. important features were permuted on held-out validation data, and the decrease in macro-F1 score was used to estimate the effect of each feature on predictive performance.

**Table 5:** Representative permutation-based feature faithfulness results.

Model	Fold	Permuted feature	Baseline macro-F1	Permuted macro-F1	Macro-F1 drop
Decision Tree	2	v_energy	1.000000	0.513055	0.486945
Decision Tree	2	h_abs_mean	1.000000	0.660446	0.339554
Decision Tree	2	h_q75	1.000000	0.717513	0.282487
Decision Tree	2	v_min	1.000000	0.773489	0.226511
Decision Tree	2	h_mean	1.000000	0.851204	0.148796
XGBoost	5	h_min	0.999878	0.998395	0.001483
XGBoost	5	hv_mean_diff	0.999878	0.998479	0.001400
CatBoost	4	h_abs_mean	1.000000	0.999228	0.000772
CatBoost	4	v_min	1.000000	0.999470	0.000530
Random Forest	1	h_q75	1.000000	1.000000	0.000000

The permutation-based faithfulness analysis showed that the Decision Tree model was highly sensitive to several individual EOG features, especially v_energy, h_abs_mean, h_q75, v_min, and h_mean. Perturbing these features caused clear decreases in macro-F1 score, supporting their importance for the model’s predictions. In contrast, CatBoost, XGBoost, and Random Forest showed very small or no performance decreases after single-feature permutation, suggesting that the ensemble models relied on redundant and complementary amplitude-related features rather than depending on one isolated feature.

## 4. Discussion

This study presented an interpretable EOG-based gaze-classification framework using window-level signal features, subject-wise validation, leakage diagnostics, and quantitative explainability analysis. Model performance was evaluated on held-out participants rather than randomly mixed time points. Under this stricter validation design, all four tree-based models achieved very high classification performance, with Random Forest showing the strongest overall results. These findings indicate that the nine gaze classes in the UNIOVI-EOG dataset are highly separable when stable 31 window-level horizontal and vertical EOG features are used.

EOG signals are temporally dependent, and adjacent samples cannot be considered fully independent observations. Therefore, classifying individual time points may lead to overoptimistic results, especially when samples from the same participant or recording session are randomly distributed across training and validation sets. By constructing 100 ms windows and retaining only windows with at least 90% label purity, the present analysis focused on stable gaze states and reduced the influence of transition periods between gaze targets [31]. This design is more consistent with practical gaze recognition, where gaze direction is usually inferred from sustained signal patterns rather than isolated instantaneous amplitudes.

The subject-wise validation and diagnostic analyses further support the credibility of the results. Participant identifiers were used only for fold construction and auditing, while all participant, session, time-order, and label-purity metadata were excluded from the model feature matrix. The absence of subject overlaps between training and validation folds reduced the possibility that the models were learning participant-specific patterns shared across random splits. In addition, the shuffled-label sanity check produced near-chance performance, indicating that the pipeline did not preserve high accuracy when the relationship between EOG features and gaze labels was disrupted. The time/order-only baseline achieved higher-than-chance but clearly lower performance than the EOG-feature-based models, suggesting that protocol order alone was not sufficient to explain the results.

The very high classification performance should be interpreted in the context of the controlled experimental design and the physiological characteristics of EOG signals. Horizontal and vertical eye movements produce systematic changes in the corresponding EOG channels, and the nine gaze classes in this dataset are defined by combinations of horizontal and vertical direction labels. The perfect performance of the simple h_mean/v_mean baseline confirms that mean horizontal and vertical amplitude values already contain strong discriminative information. Therefore, the high performance of the tree-based models is not necessarily evidence of excessive model complexity but rather reflects the strong separability of the controlled window-level EOG feature space. Nevertheless, the objective of this study was not to outperform a simple amplitude-based classifier, but rather to investigate whether explainable machine learning methods can provide transparent and physiologically meaningful insights within a leakage-controlled evaluation framework. However, this result should not be generalized directly to uncontrolled real-world environments, where motion artifacts, electrode displacement, fatigue, calibration drift, and spontaneous eye movements may reduce separability.

The comparison among models showed only minor performance differences because all classifiers were able to exploit the highly separable feature space. Random Forest achieved the best overall performance, while Decision Tree, XGBoost, and CatBoost also produced near-perfect results. In this setting, the small differences between models should not be overinterpreted as evidence of a large algorithmic advantage. Instead, the results suggest that physiologically meaningful window-level features are the main factor driving classification performance. This is also supported by the simple mean-amplitude baseline, which showed that the essential class-discriminative structure was already present in the two primary EOG channels.

The SHAP analysis provided physiologically meaningful insight into model behavior. The most influential features for the Random Forest model were mainly amplitude-level descriptors, including mean, median, quantile, and absolute mean features from the horizontal and vertical EOG channels. In contrast, variability-related and cross-channel correlation features contributed less strongly. This pattern suggests that the models primarily relied on sustained amplitude displacement rather than short-term signal variability. Such a finding is consistent with the expected behavior of EOG-based gaze recognition, where lateral gaze positions are mainly reflected in horizontal-channel changes and vertical gaze positions are mainly reflected in vertical-channel changes. Diagonal gaze classes showed combined dependence on both channels, which is also consistent with the two-dimensional structure of the class labels.

The use of both SHAP and LIME allowed the analysis to include global and local interpretability perspectives. The rank-correlation results showed strong agreement between SHAP and LIME for the ensemble models, particularly Random Forest and CatBoost, and moderate-to-strong agreement for XGBoost. This indicates that, for these models, the features identified as globally important by SHAP were generally consistent with the features contributing to local LIME explanations. However, the Decision Tree model showed weak and non-significant SHAP–LIME agreement, suggesting that explanation consistency may depend on model structure and stability. Therefore, the present findings support the use of multiple explanation methods rather than relying on a single XAI approach.

The permutation-based faithfulness analysis provided additional insight into the role of important features. For the Decision Tree model, permuting key amplitude- and energy-related features produced substantial reductions in macro-F1 score, confirming that the model depended strongly on these features. In contrast, the ensemble models showed smaller performance drops after single-feature permutation. This should not be interpreted as evidence that the features were unimportant. Rather, it likely reflects redundancy among correlated window-level features, such as mean, median, quantiles, and absolute mean amplitude. When several related features carry similar gaze information, ensemble models may remain accurate even after one feature is perturbed. Thus, permutation results should be interpreted together with SHAP and LIME rather than as a standalone explanation measure.

This study has several limitations. First, the analysis was based on a single public dataset with nine participants, which limits the ability to claim broad population-level generalization. Second, the recordings were obtained under controlled experimental conditions, and the results may not directly represent performance in real-world assistive interfaces. Third, the models were evaluated using fixed hyperparameters rather than nested hyperparameter optimization. Fourth, although SHAP–LIME agreement and permutation analysis improved the quantitative evaluation of explanations, there is no absolute ground truth for feature attribution in this task. Future work should validate the framework on external EOG datasets, larger participant groups, cross-session recordings, and more realistic online gaze-control scenarios. Additional studies should also examine the effect of different window sizes, calibration strategies, and noise conditions.

Overall, the findings show that subject-wise, window-level EOG gaze classification can achieve highly accurate and physiologically interpretable results in a controlled dataset. The combination of leakage-controlled validation, sanity-check diagnostics, SHAP, LIME, and permutation analysis strengthens the reliability of the framework. At the same time, the results should be interpreted as evidence of strong separability under controlled conditions rather than proof of unrestricted real-world robustness.

## 5. Conclusion

This study proposed an interpretable machine learning framework for EOG-based gaze classification using subject-wise validation, window-level feature extraction, and complementary explainability analyses. The methodology was designed to avoid random time-point splitting, excluding participant and temporal metadata from model inputs, and evaluate performance on held-out subjects. The results demonstrated that the nine gaze classes in the UNIOVI-EOG dataset were highly separable using physiologically meaningful horizontal and vertical EOG features, with all evaluated tree-based models achieving strong classification performance. The very high classification performance is consistent with the controlled fixation protocol and the strong separability of horizontal and vertical EOG amplitude patterns, as further supported by the simple mean-amplitude baseline and leakage diagnostic analyses. SHAP and LIME analyses showed that model decisions were mainly driven by amplitude-related features consistent with the directional characteristics of EOG signals, while quantitative agreement and faithfulness analyses provided additional support for the interpretability of the framework. The main contribution of this work lies in combining leakage-controlled subject-wise evaluation with window-level EOG feature extraction and quantitative XAI validation, providing a more transparent and methodologically robust approach to EOG-based gaze classification. Future work should extend this framework to larger datasets, cross-session recordings, and real-time assistive interface scenarios to further evaluate its generalizability and practical robustness.
